# Post-traumatic stress disorder among long-term resettled Syrian refugees in Turkey: a comprehensive analysis of pre- and post-migratory factors

**DOI:** 10.3389/fpsyt.2024.1352288

**Published:** 2024-07-02

**Authors:** Ertan Yilmaz, Lut Tamam, Cengiz Cengisiz

**Affiliations:** ^1^ Tayfur Ata Sokmen Faculty of Medicine, Department of Psychiatry, Mustafa Kemal University, Hatay, Türkiye; ^2^ Faculty of Medicine, Department of Psychiatry, Çukurova University, Adana, Türkiye; ^3^ Department of Psychiatry, Ministry of Health Manisa Mental Health and Diseases Hospital, Manisa, Türkiye

**Keywords:** Syrian refugees, Turkey, PTSD, pre-migration trauma, post-migration difficulties, mental Health

## Abstract

**Introduction:**

After the war in Syria, many people were forcibly displaced, and many others migrated to foreign countries. Many Syrians have been exposed to traumatic negative lifeexperiences during this process. In this context, this study was carried out to investigate the effects of pre- and post-migration traumatic experiences and living difficulties on the development of post-traumatic stress disorder (PTSD) in Syrian refugees who have been residing in Turkey for more than five years.

**Methods:**

The sample size of this cross-sectional study consisted of 200 Syrian refugees. Research data were collected using a self-report questionnaire. Refugees’ depression and anxiety levels were assessed with The Hopkins Symptom Checklist-25 (HSCL-25), and post-traumatic stress disorder (PTSD) symptoms were assessed with the PTSD Checklist for the Diagnostic and Statistical Manual of Mental Disorders, Fifth Edition (DSM-5) (PCL-5). Logistic regression models were created to assess the effects of pre- and post-migration traumas, adverse events, and other sociodemographic variables, including age and gender, on PTSD.

**Results:**

The study unveiled a high prevalence of PTSD (55.5%), depression (33.5%), and anxiety(4.5%) among participants. Notably, male refugees and those exposed to armed conflict exhibited a significantly higher frequency of PTSD. In contrast, depression was more prevalent among female participants. Pre-migration traumatic experiences, especially near-death situations, were identified as significant predictors of PTSD. Interestingly, while pre-migration traumatic experiences were higher, post-migration living difficulties also emerged as a concern, with factorslike "inability to return home in emergencies" and "worries about losing ethnic identity" beinghighlighted. Path analysis further revealed that pre-migration traumatic experiences indirectly contributed to PTSD by exacerbating post-migration living difficulties.

**Discussion:**

Syrian refugees in Turkey, even after long-term residence, exhibit high rates of PTSD, depression, and anxiety. While pre-migration traumas play a pivotal role, post-migration challenges further compound their mental health issues. These findings underscore the need for holistic, long-term mental health interventions that address both past traumas and current living difficulties.

## 1 Introduction

The number of forcibly displaced people worldwide exceeded 108 million by the end of 2022. Approximately 35 million people live as refugees in host countries (1). The major humanitarian crisis caused by the civil war that started in Syria in 2011 caused millions of people to be internally displaced and an increasing number of people to flee their countries. The majority of Syrians who left their homeland sought refuge in neighboring countries such as Turkey, Jordan, and Lebanon (2). According to official data, Turkey hosts approximately 3 million refugees, making it one of those countries with the largest number of refugees in the world as of 2022 (3). Turkish Government does not officially recognize those who migrated from Syria as refugees. In order to resolve the related uncertainties and improve the conditions of the refugees, an open-door policy was implemented, and temporary protection status was granted to Syrians (4). Although Turkey does not officially recognize Syrians in the country as refugees, in order to be consistent with the literature, in this article, Syrians in Turkey will be referred to as refugees instead of those under temporary protection.

Two significant risk factors affecting the mental health of refugees are pre-migration traumas and post-migration stress factors (5). For this reason, in studies investigating the problems experienced by refugees, it is necessary to first identify pre- and post-migration stress factors. Potential traumatic events come to the fore before and during migration. Forced separation from family members, kidnapping, being held hostage, injuries, death of family members and close friends, torture, sexual violence, arrests, and bombardment are common traumatic experiences (6).

On the other hand, problems experienced after migration include issues related to the immigration and acceptance procedures of the host country, including delays and uncertainties experienced in relation to the refugee-related legal processes and the fear of repatriation; problems related to working conditions, including limitations in economic opportunities, unemployment, and lack of work permit; economic problems including insufficient income level, difficulty in finding food; issues related to living conditions including difficulties in accessing mental health and psychosocial support systems and educational opportunities, not being able to stay in permanent and private housing, and housing in inappropriate conditions; social isolation, loneliness, acculturation, discrimination and language barrier (7, 8).

Mental disorders are prevalent among refugees and asylum seekers. It has been reported that mental disorders are twice as common among refugees than in the labor migrants (9).

War can have a variety of effects on the mental health of refugees, including exacerbating already existing mental disorders, giving rise to new psychological problems, and mental problems caused by various difficulties and adaptation problems experienced in the country of resettlement in the post-migration period (10).

The disorders most commonly seen in refugees are depression, anxiety, and post-traumatic stress disorder (PTSD) (11, 12). The early studies on war victims were based on the war exposure model, which focuses on trauma and related problems. However, the number of studies emphasizing that post-migration factors are as important as pre-migration factors in developing mental disorders is increasing (11, 13, 14). The post-migration period may either alleviate or exacerbate the adverse effects of trauma and related factors (15). In the post-migration period, finding a job in the host country, getting opportunities to adapt to the host society, and accessing psychosocial support systems can reduce the effects of traumatic stress on refugees (16, 17).

Considering that Turkey hosts the highest number of Syrian refugees in the world, the mental health of these refugees stands out as an important research topic. There are various studies on Syrian refugees who migrated to Turkey. Some of the studies focused on the refugees living in camps (6, 18–20), and others on those who started living in private residences after the resettlement (21, 22). One study compared internally displaced people with refugees who migrated to Turkey (23). However, refugees who were displaced five or more than five years ago constituted only a small portion of the samples of these studies. For this reason, our knowledge about the long-term mental health of refugees living in Turkey after resettlement is quite limited. The studies on the long-term mental health of refugees in other countries reported that the risk of mental illness decreases as the time spent in the host country increases (11, 12). These findings bring to mind the following research questions: “Does the effect of traumatic stress decrease in the long term (5 years or more) after resettlement?” and “Does the post-migration period put an additional burden on traumatic stress?”. Answering these questions requires investigating PTSD and related factors in refugees, taking into account post-migration stress factors as well as pre-migration traumatic experiences.

In light of this information, the objective of the study is to investigate the effects of pre- and post-migration stress factors on PTSD in Syrian refugees in the long term (5 years or more) after being resettled in Turkey.

## 2 Materials and methods

### 2.1 Research design and study sample

This cross-sectional study was conducted with adult Syrian refugees living in Adana province between October 2022 and February 2023. Adana is the 7^th^ most populated city in the country, located in the south of Turkey. According to official data, the total population of Adana in 2022 was 2 million 274 thousand 106 people, while there were 239 thousand 332 Syrian refugees. Adana, where approximately 10% of the population consists of Syrian refugees, is the 5^th^ city with the highest Syrian refugee population in Turkey (3, 24). Due to the fact that Syrian refugees were changing their addresses frequently, convenience sampling could not be performed; instead, the non-probability (snowball) sampling method was used. To this end, five neighborhoods where primarily Syrian refugees reside were identified. Efforts were made to reach all Syrian refugees living in these neighborhoods. Syrian refugees’ residential addresses were obtained from the headmen and local opinion leaders. Only one person from each address was interviewed. In addition, in cooperation with health institutions, we contacted Syrians who applied to health institutions and tried to reach other Syrian refugees living outside these five neighborhoods with the highest Syrian refugee populations.

The interviewers who conducted the interviews with Syrian refugees were university graduate translators authorized by the Ministry of Health who were fluent in Turkish in addition to their native language, i.e., Arabic. The language of the survey was Arabic. Before the interview, the translators evaluated whether the language used in the survey questions was understandable to Syrians.

Prior to conducting the interviews, the translators had been trained on the purpose of the research, the features of the survey forms and how to fill them in, and ethical considerations. The forms were filled out by the interviewed Syrian refugees. In the event that the interviewed Syrian refugees could not understand the survey questions or were illiterate, the interviewers helped the refugees fill out the survey. It took an average of 90 minutes to answer the survey. Written and verbal informed consent forms were obtained from the interviewed Syrian refugees before conducting the survey. They were assured that their data to be used within the scope of the research would be used only for scientific purposes and kept confidential and that they could withdraw their consent from the study at any time without giving any reason. No payment was made to the interviewees for participating in the research.

The study protocol was approved by the Hatay Mustafa Kemal University Ethics Committee. Additionally, necessary permissions were obtained from the Adana Provincial Directorate of Migration Management, affiliated with the General Directorate of Migration Management of the Ministry of Internal Affairs.

### 2.2 Data collection tools

#### 2.2.1 Sociodemographic form

The sociodemographic form was developed by the researchers, taking into account literature data on comparable refugee studies. The form includes questions about the refugees’ age, gender, marital status, number of children, duration of education, employment status, income level, Turkish language proficiency, the time they spent as a refugee, whether they live alone or with family, the household size, whether they have relatives left behind in their country (Syria), and whether they receive regular aid.

#### 2.2.2 The Hopkins symptom checklist

HSCL was originally developed by Parloff et al. (25). The shorter version of HSCL (HSCL-25) was developed by (26). HSCL-25 consists of 25 items assessing the presence of depression and anxiety and the severity of related symptoms in the last week. The first 10 items assess anxiety symptoms, whereas the following 15 items assess depression symptoms. HSCL-25 is a 4-point [(“Not at all” (1), “a little” (2), “quite a bit” (3), and “extremely” (4)] Likert-type self-report inventory. Subscale scores are calculated by dividing the total score obtained from the anxiety subscale by 10 and by dividing the total score obtained from the depression subscale by 15. Arabic validity and reliability studies of HSCL-25 were conducted in two different samples by Mahfoud et al. and Fares et al., who determined the cut-off scores as 2.1 for the depression subscale and 2 for the anxiety subscale (27, 28).

#### 2.2.3 Post-traumatic stress disorder checklist for the diagnostic and statistical manual of mental disorders, fifth edition

PTSD checklist for the diagnostic and statistical manual of mental disorders, fifth edition (DSM-5) (PCL-5) is a self-report scale. It consists of 20 items that assess PTSD symptoms. These 20 items are categorized into four symptom clusters reflecting the DSM-5 diagnostic criteria: Re-experiencing (items 1 to 5), Avoidance (items 6 to 7), Negative alterations (items 8 to 14), and Hyperarousal (items 15 to 20). Each item is assigned a score between 0 and 4. A maximum of 80 points can be obtained from the scale (29). Arabic translation and validation studies of the scale were conducted, and the internal consistency of the scale was found to be high (Cronbach’s alpha: 0.85). An optimum PCL-5 cut-off score of 23 predicted PTSD with 82% sensitivity and 70% specificity (30).

#### 2.2.4 List of migration experiences

The List of Migration Experiences (LIMEs) was developed based on existing checklists. It assesses whether the negative migration experiences occurred before, during, and after migration. In this way, it enables the identification of repeated negative experiences. LIMEs consist of 59 items categorized in 11 subscales. Four of these subscales, i.e., subgeneric traumas, intentional traumas, war/conflict, traumas, and worries for family members subscales, are categorized under the traumatic experiences category, and the remaining seven subscales, i.e., poverty, difficult cultural/social adaptation, difficult access to welfare facilities, problems with legal procedures, work problems, discrimination, and migration blues subscales are categorized under living difficulties category. LIMEs internal consistency (Cronbach’s alpha: 0.9) and re-test reliability (Pearson correlation for the total score 0.9, p<0.01; p at least <0.05 for any single item) were sufficiently high. Arabic translation and cultural adaptation studies of LIMEs were conducted by Aragona et al. (31).

### 2.3 Statistical analysis

The descriptive statistics obtained from the collected data were tabulated as mean ± standard deviation values in the case of continuous (numerical) variables determined to conform to the normal distribution, as median with minimum and maximum values in the case of continuous (numerical) variables determined not to conform to the normal distribution, and as numbers (n) and percentage (%) values in the case of categorical variables. Normality assumptions of the numerical variables were tested with Shapiro-Wilk, Kolmogorov-Smirnov, and Anderson-Darling tests.

In comparisons of differences between categorical variables according to groups, Pearson’s chi-square test was used in 2x2 tables with expected cells of 5 or more, Fisher’s exact test was used in tables with expected cells of less than 5, and Fisher-Freeman-Halton test was used in RxC tables with expected cells of less than 5.

In comparisons of two independent groups, the Mann-Whitney U test was used in cases where numerical variables did not conform to normal distribution.

In statistical comparisons between pre- and post-migration traumatic experiences and living difficulty variables, the Wilcoxon test was used in cases where numerical variables did not conform to normal distribution.

Logistic regression models were created to identify the variables, including pre- and post-migration traumas and other sociodemographic variables, such as age and gender, that significantly predict the development of PTSD. Each model analyzed relationships between specified variables and helped identify the variables significantly related to the development of PTSD. Relationships between independent variables were also evaluated. None of the variables had a variance inflation factor (VIF) value above 10.

Structural equation modeling (SEM) was used to examine the relationships between pre- and post-migration traumatic experiences and living difficulties. Path analysis was used to identify the direct and indirect effects of pre- and post-migration traumatic experiences and living difficulties. Path coefficients are presented as standardized coefficients. Fit indices such as the ratio of the chi-square statistic to the respective degrees of freedom (*x* ^2/sd^), goodness-of-fit index (GFI), comparative fit index (CFI), and root mean square error of approximation(RMSEA) were used to evaluate the compatibility of the model with the data.

“The statistical analyses were conducted using Jamovi project (version 2.3.28, 2023) and JASP (version 0.17.3, 2023). Both software tools were retrieved from their respective websites: Jamovi from https://www.jamovi.org and JASP from https://jasp-stats.org.”.

Additionally, path analysis was conducted using the Amos software package (trial version 26.0, IBM SPSS, Chicago, 2019). Probability (p) statistics of ≤ 0.05 were deemed to indicate statistical significance.

## 3 Results

### 3.1 Sample characteristics

The mean age of the 200 Syrian refugees living in Turkey included in the study was 36.7 ± 11.0 years. Of these refugees, 176 (88%) were married, 127 (63.5%) were male and 73 (36.5%) were female. The median number of children of the participants was 3, and the median duration of education was 5 years. The number of participants living with their families was 194 (97%). The median household size was 5. Approximately half of the participants had relatives remaining in Syria.

Of the participants, 110 (55%) had regular jobs, 173 (86.5%) had a monthly income above the minimum wage, and 10 (5%) stated that they did not have a regular monthly income. The number of participants who received regular aid was 120 (60%). The number of participants who stated they knew sufficient Turkish was 116 (58%). Lastly, the number of participants who indicated they were exposed to armed conflict was 52 (26%) (**Table 1**).

**Table 1:** Demographic characteristics of Syrian refugees living in Turkey.

	Study Group (n=200)
Age (mean ± SD, years)	36.7 ± 11.0
Gender (n, %)	
Male	127 (63.5)
Female	73 (36.5)
Marital status (n, %)	
Married	176 (88.0)
Single	18 (9.0)
Widowed	3 (1.5)
Divorced/Separated	3 (1.5)
Number of children (median and min-max)	3.0 [0.0 – 9.0]
Duration of education (median and min-max, years)	5.0 [0.0 – 16.0]
Living alone or with family (n, %)	
Alone	6 (3.0)
With family	194 (97.0)
Size of household (median and min-max)	5.0 [0.0 – 15.0]
Relatives left behind in Syria, (yes, n, %)	102 (51.0)
Regularly employed (yes, n, %)	110 (55.0)
Monthly income (n, %)	
No regular monthly income	10 (5.0)
Regular monthly income below the minimum wage	17 (8.5)
Regular monthly income above the minimum wage	173 (86.5)
Receiving regular aid from the state (yes, n, %)	120 (60.0)
Exposed to armed conflict (yes, n, %)	52 (26.0)
Proficiency in Turkish (yes, n, %)	116 (58.0)

Descriptive statistics were given as number (percentage) for categorical variables, mean ± standard deviation and median [min.-max.] for numerical variables.

The prevalence of PTSD, depression, and anxiety among the participants was 55.5%, 33.5%, and 4.5%, respectively.

A comparison of the demographic characteristics of Syrian refugees living in Turkey according to the diagnosis of PTSD did not reveal any significant difference between participants with and without PTSD in terms of age, marital status, number of children, duration of education, having been living alone or with family, size of household, having left relatives behind in Syria, monthly income, having been receiving regular assistance and sufficient knowledge of Turkish (p>0.05 for each case). However, the frequency of PTSD was found to be significantly higher in male participants than in female participants (p=0.038), in those with regular jobs than in those without regular jobs(p=0.016), and in those who were exposed to armed conflict compared to those who were not exposed to armed conflict (p=0.031). On the other hand, depression was found to be significantly higher in female participants than in male participants (p=0.028). There was no other significant difference between patients with and without depression and anxiety in terms of other demographic variables (p>0.05 for each case) (**Table 2**).

**Table 2:** Comparison of demographic characteristics of Syrian refugees living in Turkey according to the PTSD, anxiety and depression diagnoses.

	PTSD		*p* value	Anxiety		*p* value	Depression		*p* value
	No (n=89)	Yes (n=111)		No (n=191)	Yes (n=9)		No (n=133)	Yes (n=67)	
Age (median and min-max, years)	35.0 [20.0 – 82.0]	35.0 [20.0 – 66.0]	0.314**	35.0 [20.0 – 82.0]	37.0 [25.0 – 51.0]	0.941**	35.0 [20.0 – 82.0]	35.0 [20.0 – 65.0]	0.202**
Gender (n, %)									
Male	49 (55.1)	78 (70.3)	0.038*	123 (64.4)	4 (44.4)	0.291*	92 (69.2)	35 (52.2)	0.028*
Female	40 (44.9)	33 (29.7)		68 (35.6)	5 (55.6)		41 (30.8)	32 (47.8)	
Marital status (n, %)									
Married	75 (84.3)	101 (91.0)	0.548*	168 (88.0)	8 (88.9)	0.664*	116 (87.2)	60 (89.6)	0.883*
Single	10 (11.2)	8 (7.2)		17 (8.9)	1 (11.1)		12 (9.0)	6 (9.0)	
Widowed	2 (2.2)	1 (0.9)		3 (1.6)	0 (0.0)		3 (2.3)	0 (0.0)	
Divorced/Separated	2 (2.2)	1 (0.9)		3 (1.6)	0 (0.0)		2 (1.5)	1 (1.5)	
Number of children (Median and min-max)	3.0 [0.0 – 9.0]	3.0 [0.0 – 8.0]	0.963**	3.0 [0.0 – 9.0]	3.0 [0.0 – 5.0]	0.827**	3.0 [0.0 – 9.0]	3.0 [0.0 – 7.0]	0.392**
Duration of education (Median and min-max, years)	5.0 [0.0 – 16.0]	5.0 [0.0 – 16.0]	0.322**	5.0 [0.0 – 16.0]	0.0 [0.0 – 12.0]	0.362**	5.0 [0.0 – 16.0]	5.0 [0.0 – 16.0]	0.649**
Living alone or with family (n, %)									
Alone	4 (4.5)	2 (1.8)	0.410*	5 (2.6)	1 (11.1)	0.244*	2 (1.5)	4 (6.0)	0.098*
With family	85 (95.5)	109 (98.2)		186 (97.4)	8 (88.9)		131 (98.5)	63 (94.0)	
Size of household (Median and min-max)	5.0 [1.0 – 9.0]	5.0 [0.0 – 15.0]	0.817**	5.0 [0.0 – 15.0]	5.0 [1.0 – 11.0]	0.688**	5.0 [1.0 – 15.0]	5.0 [0.0 – 11.0]	0.412**
Relatives left behind in Syria, (*Yes, n, %)*	40 (44.9)	62 (55.9)	0.164*	98 (51.3)	4 (44.4)	0.744*	71 (53.4)	31 (46.3)	0.424*
Regularly employed (yes, n, %)	40 (44.9)	70 (63.1)	0.016*	107 (56.0)	3 (33.3)	0.304*	80 (60.2)	30 (44.8)	0.056*
Monthly income (n, %)									
None	6 (6.7)	4 (3.6)	0.620*	9 (4.7)	1 (11.1)	0.135*	8 (6.0)	2 (3.0)	0.759*
Less than 2000	7 (7.9)	10 (9.0)		15 (7.9)	2 (22.2)		11 (8.3)	6 (9.0)	
More than 2000	76 (85.4)	97 (87.4)		167 (87.4)	6 (66.7)		114 (85.7)	59 (88.1)	
Receiving regular aid from the state (*Yes, n, %)*	58 (65.2)	62 (55.9)	0.234*	116 (60.7)	4 (44.4)	0.488*	80 (60.2)	40 (59.7)	0.999*
Exposed to armed conflict (yes, n, %)	16 (18.0)	36 (32.4)	0.031*	50 (26.2)	2 (22.2)	0.999*	29 (21.8)	23 (34.3)	0.083*
Proficiency in Turkish (yes, n, %)	47 (52.8)	69 (62.2)	0.235*	111 (58.1)	5 (55.6)	0.999*	81 (60.9)	35 (52.2)	0.308*

Descriptive statistics were given as number (percentage) for categorical variables, median [min.-max.] for numerical variables.

*. Pearson Chi-Square, Fisher’s Exact or Fisher-Freeman-Halton tests.

**. Mann-Whitney U test.

Bold values indicate statistical significance.

It was determined that the most common pre- and post-migration traumatic experiences among Syrian refugees living in Turkey were “damage to personal belongings” and “inability to return home in an emergency situation”, which were experienced by 166 (83%) and 64 (32%) participants, respectively. Additionally, the most common pre- and post-migration living difficulties experienced by Syrian refugees living in Turkey were “feeling of injustice done to self” and “cultural adaptation and coping difficulties”, which were experienced by 149 (74.5%) and 139 (69.5%) participants, respectively. Traumatic experiences and living difficulties experienced by refugees are shown in **Table 3**.

**Table 3:** Comparison of the 10 most common pre- and post-migration traumatic experiences and living difficulties experienced by Syrian refugees living in Turkey according to PTSD diagnosis.

	Study Group (n=200)	PTSD		*p* value*
		No PTSD (n=89)	PTSD (n=111)	
Pre-Migration				
Traumatic Experiences				
Damage to personal belongings	166 (83)	75 (84.3)	91 (82.0)	0.811
Murder of a family member or friend	129 (64.5)	54 (60.7)	75 (67.6)	0.388
Having been attacked	101 (50.5)	37 (41.6)	64 (57.7)	**0.034**
Having witnessed violence against others	93 (46.5)	39 (43.8)	54 (48.6)	0.591
Unnatural death of a family member or friend	76 (38)	33 (37.1)	43 (38.7)	0.925
Having had an accident	71 (35.5)	25 (28.1)	46 (41.4)	0.070
Loss or separation of family members	55 (27.5)	21 (23.6)	34 (30.6)	0.343
Forced separation from family members	45 (22.5)	14 (15.7)	31 (27.9)	0.060
Having experienced a near-death situation	38 (19)	8 (9.0)	30 (27.0)	**0.002**
Murder of one (more than one) stranger	23 (11.5)	11 (12.4)	12 (10.8)	0.906
Living Difficulties				
Feeling of injustice done to self	149 (74.5)	61 (68.5)	88 (79.3)	0.117
Scarcity of food and water	107 (53.5)	47 (52.8)	60 (54.1)	0.974
Lack of access to favorite foods	104 (52)	46 (51.7)	58 (52.3)	0.999
Overcrowded accommodation	50 (25)	18 (20.2)	32 (28.8)	0.218
Insufficient well-being aid from the state	40 (20)	14 (15.7)	26 (23.4)	0.240
Feeling of being neglected	29 (14.5)	12 (13.5)	17 (15.3)	0.870
Inadequate access to counseling services	27 (13.5)	12 (13.5)	15 (13.5)	0.999
Insufficient well-being aid from charitable organizations	25 (12.5)	9 (10.1)	16 (14.4)	0.484
Feeling of not deserving of living as an immigrant	20 (10)	12 (13.5)	8 (7.2)	0.218
Lack of accommodation	17 (8.5)	6 (6.7)	11 (9.9)	0.587
Post-Migration				
Traumatic Experiences				
Inability to return home in an emergency situation	64 (32)	22 (24.7)	42 (37.8)	0.068
Concerns about the relatives left behind in Syria	43 (21.5)	14 (15.7)	29 (26.1)	0.108
Forced separation from family members	2 (1)	2 (2.2)	0 (0.0)	0.197
Living Difficulties				
Difficulties adapting and coping with the culture	139 (69.5)	66 (74.2)	73 (65.8)	0.260
Feeling of being a minority	136 (68)	64 (71.9)	72 (64.9)	0.363
Loneliness and boredness	122 (61)	57 (64.0)	65 (58.6)	0.519
Difficulties associated with language differences	120 (60)	54 (60.7)	66 (59.5)	0.977
Feeling of not being able to control the events in personal life	120 (60)	59 (66.3)	61 (55.0)	0.139
Feeling of being deprived	119 (59.5)	57 (64.0)	62 (55.9)	0.304
Concerns about own culture	118 (59)	59 (66.3)	59 (53.2)	0.083
Feeling of being neglected	114 (57)	50 (56.2)	64 (57.7)	0.947
Not being allowed to work	113 (56.5)	47 (52.8)	66 (59.5)	0.424
Worrying about losing ethnic identity	113 (56.5)	58 (65.2)	55 (49.5)	**0.038**

Descriptive statistics were given as numbers (percentages) for categorical variables.

*Pearson Chi-Square or Fisher’s Exact tests.

Bold values indicate statistical significance.

The mean number of traumatic experiences experienced in the pre-migration period was significantly higher than in the post-migration period (p<0.001 for each case). On the contrary, the mean number of living difficulties experienced in the post-migration period was found to be significantly higher than in the pre-migration period (p<0.001 for each case) except for poverty (p<0.001). There was no significant difference between the pre- and post-migration periods in terms of difficulties in accessing welfare facilities (p=0.263).

Univariate and multivariate analyses revealed that a one-fold increase in pre-migration traumatic experience increased the risk of developing PTSD by 1.11 times (p = 0.044). On the other hand, while female gender was found to increase the risk of PTSD by 0.52 times in univariate analyses, no significant relationship was found between gender and PTSD risk in multivariate analyses (p = 0.839) (**Table 4**). Univariate analyses did not reveal any significant effect of post-migration traumatic experiences and living difficulties and pre-migration living difficulties on the development of PTSD (p>0.05 for each case). Multivariate analyses revealed that a one-fold increase in pre-migration traumatic experience increased the risk of developing PTSD by 1.11 times (p=0.045) and that post-migration traumatic experiences did not have any significant effect on the risk of developing PTSD (p=0.057) (**Table 5**).

**Table 4:** The effects of age, gender, pre-migration traumatic experiences, and post-migration living difficulties on the development of PTSD in Syrian refugees living in Turkey.

	Univariate LR		Multivariate LR	
	OR [%95 CI]	*p* value	OR [%95 CI]	*p* value
Pre-Migration Traumatic Experiences	1.11 [1.01 – 1.22]	0.044	1.11 [1.01 – 1.22]	**0.050**
Post-Migration Living Difficulties	1.01 [0.97 – 1.06]	0.521	–	–
Age	1.01 [0.98 – 1.03]	0.616	–	–
Gender: *Female* vs. *Male*	0.52 [0.29 – 0.93]	0.027	1.00 [0.98 – 1.03]	0.839

LR, Logistic regression; OR, Odds ratio; CI, Confidence interval.

Bold values indicate statistical significance.

**Table 5:** The effects of pre-migration traumatic experiences and post-migration living difficulties on the development of PTSD in Syrian refugees living in Turkey.

	Univariate LR		Multivariate LR	
	OR [%95 CI]	*p* value	OR [%95 CI]	*p* value
Post-Migration Traumatic Experiences	1.43 [0.99 – 2.08]	0.057	1.44 [0.99 – 2.1]	0.057
Post-Migration Living Difficulties	1.01 [0.97 – 1.06]	0.521		
Pre-Migration Traumatic Experiences	1.11 [1.01 – 1.22]	0.044	1.11 [1.01 – 1.23]	**0.045**
Pre-Migration Living Difficulties	1.11 [0.98 – 1.26]	0.107		

LR, Logistic regression; OR, Odds ratio; CI, Confidence interval.

Bold values indicate statistical significance.

Univariate analysis of the ten most common pre-migration traumatic experiences revealed that the risk of developing PTSD increased by 1.91 times if “having been attacked” (p=0.024), by 1.81 times if “having had an accident” (p=0.051), by 2.08 times if “forcibly separated from family members” (p=0.042) and by 3.75 times (p=0.002) if experienced a “near-death situation”. Other most common pre-migration traumatic experiences had no significant effect on the development of PTSD (p>0.05 for each case). Additionally, univariate analyses revealed that none of the ten most common pre-migration living difficulties significantly affected the development of PTSD (p>0.05 for each case).

Multivariate analyses revealed that only experiencing a “near-death situation” among the pre-migration traumatic experiences significantly (2.8 times) increased the risk of developing PTSD(p=0.027) and that none of the ten most common pre-migration living difficulties had a significant effect on the development of PTSD (p>0.05 for each case) (**Table 6**).

**Table 6:** The effects of the 10 most common pre-migration traumatic experiences and living difficulties on the development of PTSD in Syrian refugees living in Turkey.

	Univariate LR		Multivariate LR	
	OR [%95 CI]	*p* value	OR [%95 CI]	*p* value
Pre-Migration				
Traumatic Experiences				
Damage to personal belongings: *Yes* vs. *No*	0.85 [0.40 – 1.79]	0.669		
Murder of a family member or friend: *Yes* vs. *No*	1.35 [0.75 – 2.42]	0.312		
Having been attacked: *Yes* vs. *No*	1.91 [1.09 – 3.37]	**0.024**	1.29 [0.66 – 2.5]	0.458
Having witnessed violence against others: *Yes* vs. *No*	1.21 [0.69 – 2.13]	0.496		
Unnatural death of a family member or friend: *Yes* vs. *No*	1.07 [0.60 – 1.91]	0.810		
Having had an accident: *Yes* vs. *No*	1.81 [1.00 – 3.29]	0.051	1.16 [0.58 – 2.34]	0.669
Loss or separation of family members: *Yes* vs. *No*	1.43 [0.76 – 2.70]	0.269		
Forced separation from family members: *Yes* vs. *No*	2.08 [1.03 – 4.20]	**0.042**	1.64 [0.79 – 3.44]	0.187
Having experienced a near-death situation: *Yes* vs. *No*	3.75 [1.62 – 8.67]	**0.002**	2.80 [1.12 – 7.01]	**0.027**
Murder of one (more than one) stranger: *Yes* vs. *No*	0.86 [0.36 – 2.05]	0.733		
Living Difficulties				
Feeling of injustice done to self: *Yes* vs. *No*	1.76 [0.93 – 3.33]	0.085	1.88 [0.95 – 3.7]	0.068
Scarcity of food and water: *Yes* vs. *No*	1.05 [0.60 – 1.84]	0.861		
Lack of access to favorite foods: *Yes* vs. *No*	1.02 [0.59 – 1.79]	0.936		
Overcrowded accommodation: *Yes* vs. *No*	1.60 [0.83 – 3.09]	0.164	1.78 [0.87 – 3.64]	0.114
Insufficient well-being aid from the state: *Yes* vs. *No*	1.64 [0.80 – 3.37]	0.179	1.23 [0.57 – 2.66]	0.603
Feeling of being neglected: *Yes* vs. *No*	1.16 [0.52 – 2.58]	0.715		
Inadequate access to counseling services: *Yes* vs. *No*	1.00 [0.44 – 2.27]	0.995		
Insufficient well-being aid from charitable organizations: *Yes* vs. *No*	1.50 [0.63 – 3.57]	0.363		
Feeling of not deserving of living as an immigrant: *Yes* vs. *No*	0.50 [0.19 – 1.28]	0.147	0.36 [0.13 – 0.98]	**0.045**
Lack of accommodation: *Yes* vs. *No*	1.52 [0.54 – 4.29]	0.427		

LR, Logistic regression; OR, Odds ratio; CI, Confidence interval.

Bold values indicate statistical significance.

Univariate analysis revealed that only “inability to return home in an emergency situation” among the post-migration traumatic experiences significantly (1.85 times) increased the risk of developing PTSD (p=0.049) and that no other post-migration traumatic experiences had a significant effect on the development of PTSD (p>0.05 for each case). Additionally, univariate analysis revealed that only “worrying about losing ethnic identity” among the post-migration living difficulties significantly (0.52times) increased the risk of developing PTSD(p=0.028) and that no other post-migration living difficulties had a significant effect on the development of PTSD (p>0.05 for each case).On the other hand, multivariate analyses showed that none of the post-migration traumatic experiences and living difficulties had a significant effect on the risk of developing PTSD (p>0.05 for each case) (**Table 7**).

**Table 7:** The effects of the 10 most common post-migration traumatic experiences and living difficulties on the development of PTSD in Syrian refugees living in Turkey.

	Univariate LR		Multivariate LR	
	OR [%95 CI]	*p* value	OR [%95 CI]	*p* value
Post-Migration				
Traumatic Experiences				
Inability to return home in an emergency situation: *Yes* vs. *No*	1.85 [1.01 – 3.43]	0.049	1.56 [0.75 – 3.24]	0.232
Concerns about the relatives left behind in Syria: *Yes* vs. *No*	1.89 [0.93 – 3.86]	0.078	1.44 [0.62 – 3.34]	0.400
Forced separation from family members: *Yes* vs. *No*	0.01 [0.01 – 0.02]	0.988		
Living Difficulties				
Difficulties adapting and coping with the culture: *Yes* vs. *No*	0.67 [0.36 – 1.24]	0.201	1.07 [0.46 – 2.47]	0.880
Feeling of being a minority: *Yes* vs. *No*	0.72 [0.39 – 1.32]	0.289		
Loneliness and boredness: *Yes* vs. *No*	0.79 [0.45 – 1.41]	0.430		
Difficulties associated with language differences: *Yes* vs. *No*	0.95 [0.54 – 1.68]	0.862		
Feeling of not being able to control the events in personal life: *Yes* vs. *No*	0.62 [0.35 – 1.10]	0.105	1.01 [0.44 – 2.33]	0.975
Feeling of being deprived: *Yes* vs. *No*	0.71 [0.40 – 1.26]	0.242	0.85 [0.43 – 1.66]	0.629
Concerns about own culture: *Yes* vs. *No*	0.58 [0.32 – 1.03]	0.061	1.60 [0.28 – 9.12]	0.599
Feeling of being neglected: *Yes* vs. *No*	1.06 [0.60 – 1.87]	0.834		
Not being allowed to work: *Yes* vs. *No*	1.31 [0.75 – 2.30]	0.346		
Worrying about losing ethnic identity: *Yes* vs. *No*	0.52 [0.30 – 0.93]	0.028	0.34 [0.06 – 2.15]	0.254

LR, Logistic regression; OR, Odds ratio; CI, Confidence interval.

### 3.2 Path analysis results

**Figure 1:**
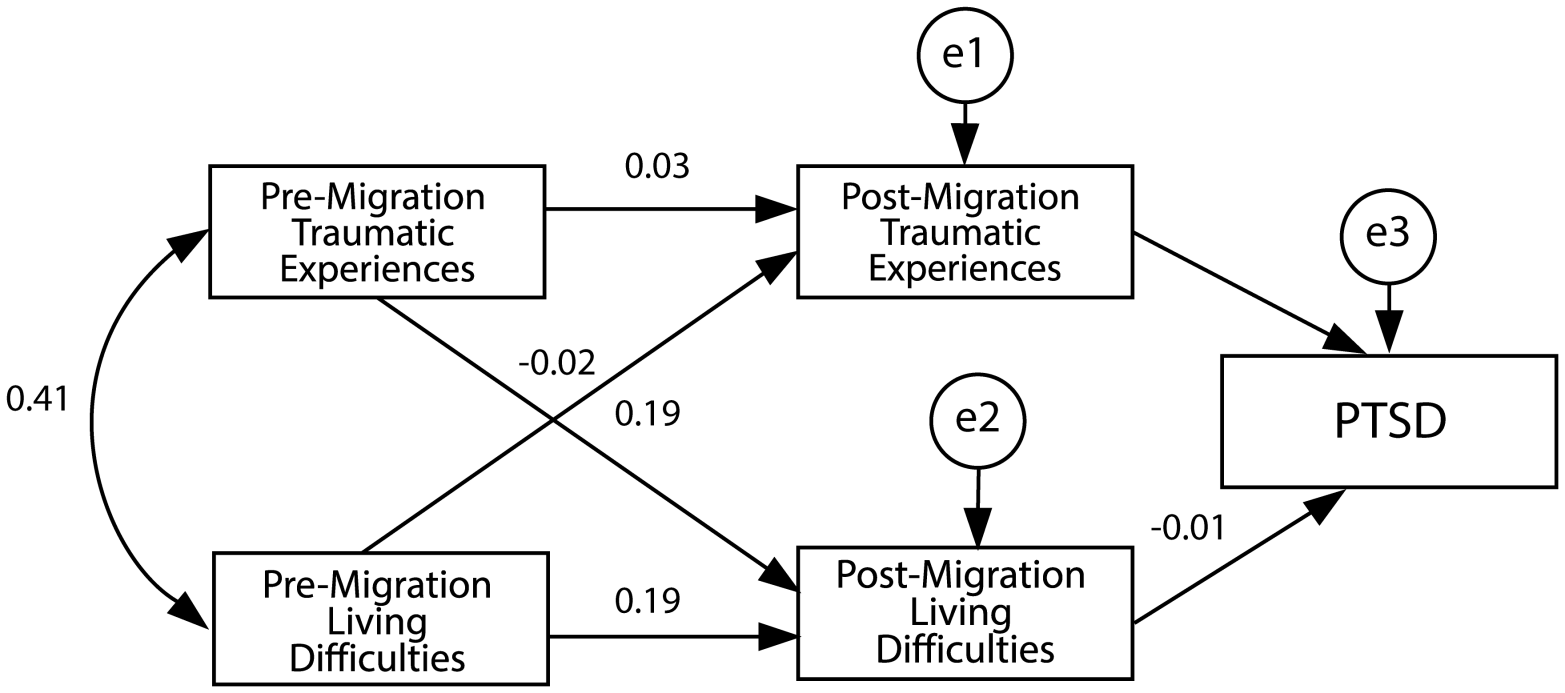
Determining the likelihood of developing PTSD. The path analysis model investigates the relationships between pre- and post-traumatic experiences. The model estimation was calculated by the Mardia test using a multivariate normal distribution of the variables.

The path analysis model was created to investigate the relationships between pre- and post-traumatic experiences, living difficulties, and the risk of developing PTSD. The multivariate normal distribution of the variables included in the said model was examined with the Mardia test. Accordingly, Mardia’s coefficient was found to be -1.399, and the minimum and maximum values of the variables, skewness, and kurtosis coefficients were given. Given that the Mardia’s coefficient was less than 3, maximum likelihood estimation was used when testing the model. Notably, the analysis of the skewness and kurtosis coefficients of the PTSD variable indicated that the PTSD variable deviated from normal distribution. These findings helped select an appropriate estimation method for investigating the relationships between pre- and post-traumatic experiences, living difficulties, and the risk of developing PTSD (**Figure 1**).

The path coefficient pertaining to the cause-and-effect relationship between pre-migration traumatic experiences and post-migration living difficulties was determined to be statistically significant (p<0.05) (**Table 8**). The standardized path coefficient pertaining to the cause-and-effect relationship between pre- and post-migration living difficulties was calculated as -0.188, indicating that a one-fold increase in pre-migration living difficulties leads to a 0.188-fold decrease in post-migration living difficulties. The path coefficient pertaining to the cause-and-effect relationship between pre-migration traumatic experiences and post-migration living difficulties was also determined to be statistically significant (p<0.05). The standardized path coefficient pertaining to the cause-and-effect relationship between pre-migration traumatic experiences and post-migration living difficulties was calculated as 0.189, indicating that a one-fold increase in pre-migration traumatic experiences leads to a 0.189-fold increase in post-migration living difficulties. Additionally, the path coefficient pertaining to the cause-and-effect relationship between post-migration traumatic experiences and the risk of developing PTSD was also statistically significant (p<0.05). The standardized path coefficient pertaining to the cause-and-effect relationship between post-migration traumatic experiences and the risk of developing PTSD was calculated as 0.139, indicating that a one-fold increase in post-migration traumatic experiences leads to a 0.139-fold increase in the risk of developing PTSD. Path coefficients pertaining to the cause-and-effect relationships between other variables were not statistically significant (p>0.05 for each case). Model fit statistics were as follows: $\chi^{2}/sd$ =12.582; GFI = 0.93; CFI=0.54 and RMSEA = 0.241. Accordingly, only the GFI index indicated the model’s compatibility with the data among the fit indices.

**Table 8:** PTSD path analysis.

			Path Coefficient	Standard Error	Standardized Path Coefficient	*p* value
Post-Migration Traumatic Experiences	←	Pre-Migration Traumatic Experiences	0.007	0.020	0.028	0.715
Post-Migration Living Difficulties	←	Pre-Migration Living Difficulties	-0.520	0.210	-0.188	**0.014**
Post-Migration Traumatic Experiences	←	Pre-Migration Living Difficulties	-0.007	0.027	-0.020	0.800
Post-Migration Living Difficulties	←	Pre-Migration Traumatic Experiences	0.397	0.160	0.189	**0.013**
PTSD	←	Post-Migration Traumatic Experiences	0.088	0.045	0.139	**0.048**
PTSD	←	Post-Migration Living Difficulties	-0.001	0.006	-0.008	0.907

The '←' symbol indicates the direction of the relationship between two variables.

The symbol denotes that the variable on the left affects the variable on the right.

Bold values indicate statistical significance.

## 4 Discussion

The prevalence of depression, anxiety, and PTSD among the Syrian refugees living in Turkey was 33.5%, 4.5%, and 55.5%, respectively. These findings demonstrated that refugees’ risk of psychiatric morbidity, PTSD in particular, remains high even long after their migration. In a review study, Bogic et al. reported that the prevalence of depression, anxiety, and PTSD in refugees five or more years after their displacement ranged from 2.3–80%, 20.3–88%, and 2.3–80%, respectively (13). A systematic review reported that the prevalence of depression, anxiety, and PTSD in Syrian refugees ranged from 30.2–50.5%, 23.2–42.6%, and 23.2–42.6%, respectively (32). Similarly, in this study, the prevalence of mental disorders and psychiatric symptoms was significantly higher in refugee groups than in the general population. In parallel, a systematic review concluded that the risk of PTSD in refugees resettled in Western countries is ten times higher than in the general population (11). The prevalence of PTSD was higher, whereas the prevalence of anxiety was lower in our study group compared to other studies on Syrian refugees. These differences between studies can be attributed to differences in the environments in which refugees are resettled after migration and the length of time that has passed since refugees’ traumatic experiences. Refugees living in permanent private housing have better mental health outcomes than those receiving temporary housing assistance. Additionally, it has been observed that the longer the time that has passed since traumatic experiences, the better the mental health outcomes (16). The mean time elapsed since the displacement of the refugees included in this study was quite long. Hence, almost all of them were living in private housing with their families at the time of the study. Yet, the prevalence of PTSD among the refugees was still high. The measurement methods used may explain some of these findings. Structured diagnostic criteria such as DSM and International Classification of Diseases (ICD) are associated with lower prevalence estimates. Prevalence estimates of depression, anxiety, and PTSD are estimated to be 1.5–2 times higher in studies using self-report surveys (33). Additionally, studies conducted in developed countries may report a lower prevalence of mental problems (9). DSM and ICD are universal diagnostic systems and may overlook some cultural characteristics. The large variability between the findings of studies on populations affected by armed conflicts makes their interpretation difficult. Another factor affecting the mental health of refugees is the construct definition of the data collection tool, that is, whether it was developed for refugee research and whether culture-specific validity and reliability studies have been conducted for the given refugee sample. The self-report tools we used in our study were PCL-5 and HSCL-25. The PCL-5 is a 20-item self-report scale that assesses the 20 DSM-5 symptoms of PTSD. These 20 items are categorized into four symptom clusters reflecting the DSM-5 diagnostic criteria: Re-experiencing, Avoidance, Negative alterations, and Hyperarousal. The HSCL-25 was found to be useful as a measurement tool for traumatized refugees speaking different languages (30, 34). The optimum cut-off score for the original version of PCL-5 has been reported as 33. In comparison, in this study, 23 was used as the optimum cut-off score, as indicated in the Arabic validity and reliability studies of PCL-5 (30). The cut-off scores we used may have contributed to the high prevalence of PTSD, which would then mean that the optimum cut-off score for the Arabic version of PCL-5 may need to be verified in additional studies.

The high rates of PTSD among Syrian refugees who have spent a long time in our country can be partially explained by cultural factors. A diagnosis of mental illness can cause stigmatization in Syrian culture, hindering patient’s attempts to seek help and treatment (8).

Psychiatric problems, especially traumatic symptoms, can be seen at high rates in the early stages of migration. In follow-up studies exceeding ten years, it has been reported that the psychiatric symptoms of refugees gradually improved. In a study by Steel et al. comparing Vietnamese refugees who took refuge in Australia with Australian-born people, refugees’ mental disorder rates were lower than local people (35). Similar results were found in a follow-up study conducted in Canada (36). Improvements in the long term have also been reported among Hmong people living in the U.S (37).. These results do not support the argument that mental problems will still largely exist after a long-term refugee experience. The initially high rates of psychiatric morbidity in refugees tend to decrease over time, especially in those exposed to low-level trauma (5). In contrast, in a study conducted with Iraqi asylum seekers who immigrated to the Netherlands, Laban et al. determined that the prevalence of psychiatric disorders was lower among the new arrivals than the ones who had stayed in the country for more than two years. They attributed this result to the lengthy asylum procedures in force in the Netherlands (38). These results suggest that post-migration factors may improve or worsen mental health.

In our study, no significant difference was found between participants with and without a diagnosis of PTSD in terms of age, marital status, number of children, duration of education, whether they live alone or with family, household size, whether they have relatives left behind in their country (Syria), monthly income, whether they receive regular aid and sufficient knowledge of Turkish.

PTSD was found to be significantly more common in male refugees, those with regular jobs, and those exposed to armed conflict. No significant relationship was found between the prevalence of PTSD and female gender. The fact that male refugees are more likely to be exposed to armed conflict may have caused them to have a higher risk of PTSD.

In line with the findings of many field studies conducted around the world, in this study, depression was found to be significantly more common in female refugees. None of the demographic characteristics significantly affected depression (39).

Another finding of our study is that the mean number of traumatic experiences experienced in the pre-migration period was significantly higher than in the post-migration period. A one-fold increase in pre-migration traumatic experience increased the risk of developing PTSD by 1.11 times. The risk of PTSD increased significantly by 2.8-fold in refugees who experienced a “near-death situation”, whereas no other pre-migration traumatic experiences had any significant effect on the development of PTSD. This finding was consistent with the results of previous studies in which pre-migration traumatic experiences were found to be a risk factor for mental disorders (40, 41). In addition to “near-death situation”, univariate analysis revealed that the risk of developing PTSD also increased if experienced an “attack situation”, if experienced an “accident”, and if “forcibly separated from family members”. However, none of these pre-migration traumatic experiences were significantly related to PTSD risk in multivariate analysis.

The mean number of post-migration traumatic experiences reported by the Syrian refugees included in our study was significantly less than that of pre-migration traumatic experiences. Among the post-migration traumatic experiences, “inability to return home in an emergency situation”, reported by 32% of the refugees, and “concerns about the relatives left behind in Syria”, reported by 21% of the refugees, came to the fore. However, neither of these two traumatic experiences was found to be significantly associated with PTSD. This finding may be attributed to the lower number of post-migration traumatic experiences compared to pre-migration traumatic experiences and the fact that, unlike pre-migration traumatic experiences, they do not involve a near-death situation. On the other hand, the mean number of post-migration living difficulties was significantly higher than that of pre-migration living difficulties. Among the living difficulties experienced during the post-migration period, it was hypothesized that “worrying about losing ethnic identity” could be a risk factor for PTSD. Although univariate analysis indicated that “worrying about losing ethnic identity” significantly increased the risk for PTSD, no significant relationship was found between “worrying about losing ethnic identity” and the risk for PTSD in multivariate analysis.

In order to evaluate whether there are correlations between pre-migration traumatic experiences, post-migration living difficulties, and the development and continuation of PTSD in refugees, the path coefficients pertaining to the cause-and-effect relationship between pre-migration traumatic experiences and post-migration living difficulties were calculated within the scope of the path analysis. Consequently, it was determined that a one-fold increase in pre-migration traumatic experiences causes an approximately 18% increase in post-migration living difficulties. Therefore, in addition to their direct impact on the development of PTSD, pre-migration traumatic experiences also indirectly contribute to the continuation of PTSD by increasing post-migration living difficulties. These findings are consistent with those of a growing number of studies indicating a relationship between post-migration factors and psychiatric morbidity (42–44). Laban et al. found that post-migration life difficulties increased the risk of psychopathology more than pre-migration life difficulties but did not increase the risk of PTSD (38). Post-migration stressors can increase the impact of traumatic events on refugees who have experienced many traumatic events, thus whose ability to cope with stress has weakened, rendering them vulnerable to new stressors (45). There is also evidence showing that post-migration events mediate between pre-migration traumatic experiences and psychological distress (46). Previous studies on refugees have been criticized for being conducted solely from a biomedical perspective, excluding the social perspective. Given that events such as war and forced displacement experienced by refugees are major events that have a profound impact on other issues related to refugees, it has been suggested that it may be more explanatory to address the mental health and adaptation of refugees through a biopsychosocial model, which includes reciprocal and causal relationships in biological, social and psychological fields, instead of a biomedical perspective (47).

In sum, this study’s findings indicated that PTSD is common among Syrian refugees living in Turkey, pre-migration traumatic experiences have a direct effect on PTSD, and post-migration living difficulties have an indirect effect.

It is known that pre-migration traumatic experiences increase the risk of psychiatric morbidities. Nevertheless, post-migration living difficulties experienced in the host country may also increase the risk of psychiatric morbidities. The fact that the refugee population lives with their families in private housing and has regular jobs may stand out as protective factors for PTSD. In addition, Syrian refugees’ access to health services, especially mental health services, is provided free of charge. However, there are some barriers in accessing mental health services. One of them is cultural reasons. The second reason is the language barrier, which makes access to mental health services very difficult. In order to increase the quality of health services and to overcome the language barrier issue, the Ministry of Health has assigned a large number of Syrian health workers in hospitals. In addition, translators fluent in both languages have been employed. However, the scarcity of centers that offer trauma-focused intervention methods is a problem. There is also a lack of approaches to prevent traumatic events at the individual, family and community scale levels. In this context, identifying post-migration living difficulties that mediate the development and continuation of psychiatric disorders, including PTSD, will enable interventions to improve the mental health of refugees.

Our study has some strengths and limitations. First, to the best of this study’s authors’ knowledge, this is the first study to date on the mental health outcomes of refugees living in Turkey for five years or more since migration. On the other hand, the fact that research data were collected through self-report surveys, not structured interviews, can be considered a limitation of the study. In our study, 4 self-report tools were used, and participants answered a very large number of questions. It took an average of 90 minutes to answer the survey. This might have affected the participants responses. The fact that some of the respondents were reached through community headmen, local opinion leaders might also have disrupted anonymity and affected the responses of the participants. The fact that some of our subjects have been contacted through health institutions may have led to the inclusion of already diagnosed individuals as another contributor to bias. Mobilization of the refugees made it difficult for us to determine the target population and use the convenience sampling method. Additionally, the majority of the refugees included in the study have been receiving regular aid from the state. This may have caused bias in answering some questions. Syrian refugees reside in almost every region of Turkey. Considering that this study was conducted in only one province (Adana), it may not be possible to generalize the study results to the whole of Turkey. Yet, the study can be considered an important study in terms of revealing the factors that contribute to the development of PTSD in refugees and guiding future studies in this field.

## Data availability statement

The original contributions presented in the study are included in the article/supplementary material. Further inquiries can be directed to the corresponding author.

## Ethics statement

The studies involving humans were approved by Hatay Mustafa Kemal University Ethics Committee (Approval Date/No: 06.10.2022/23). The studies were conducted in accordance with the local legislation and institutional requirements. The participants provided their written informed consent to participate in this study.

## Author contributions

EY: Conceptualization, Formal analysis, Investigation, Methodology, Project administration, Resources, Software, Supervision, Validation, Writing – original draft, Writing – review & editing. LT: Conceptualization, Formal analysis, Investigation, Methodology, Software, Supervision, Validation, Writing – original draft, Writing – review & editing. CC: Conceptualization, Investigation, Methodology, Resources, Supervision, Writing – original draft, Writing – review & editing.
